# Celiac Disease: A Review of Current Concepts in Pathogenesis, Prevention, and Novel Therapies

**DOI:** 10.3389/fped.2018.00350

**Published:** 2018-11-21

**Authors:** Jason A. Tye-Din, Heather J. Galipeau, Daniel Agardh

**Affiliations:** ^1^Immunology Division, The Walter and Eliza Hall Institute, Parkville, VIC, Australia; ^2^Department of Medical Biology, University of Melbourne, Parkville, VIC, Australia; ^3^Department of Gastroenterology, The Royal Melbourne Hospital, Parkville, VIC, Australia; ^4^Centre for Food & Allergy Research, Murdoch Children's Research Institute, Parkville, VIC, Australia; ^5^Farncombe Family Digestive Health Research Institute, McMaster University, Hamilton, ON, Canada; ^6^The Diabetes and Celiac Disease Unit, Department of Clinical Sciences, Lund University, Malmö, Sweden; ^7^Unit of Endocrinology and Gastroenterology, Department of Pediatrics, Skåne University Hospital, Malmö, Sweden

**Keywords:** celiac disease, gluten, T cells, microbiome, pathogenesis

## Abstract

Our understanding of celiac disease and how it develops has evolved significantly over the last half century. Although traditionally viewed as a pediatric illness characterized by malabsorption, it is now better seen as an immune illness with systemic manifestations affecting all ages. Population studies reveal this global disease is common and, in many countries, increasing in prevalence. These studies underscore the importance of specific HLA susceptibility genes and gluten consumption in disease development and suggest that other genetic and environmental factors could also play a role. The emerging data on viral and bacterial microbe-host interactions and their alterations in celiac disease provides a plausible mechanism linking environmental risk and disease development. Although the inflammatory lesion of celiac disease is complex, the strong HLA association highlights a central role for pathogenic T cells responding to select gluten peptides that have now been defined for the most common genetic form of celiac disease. What remains less understood is how loss of tolerance to gluten occurs. New insights into celiac disease are now providing opportunities to intervene in its development, course, diagnosis, and treatment.

## Introduction

Celiac disease (CeD) is a chronicimmune-mediated enteropathyprecipitated by to dietary gluten in genetically predisposed individuals (1). Current diagnosis is based on demonstrating the enteropathy in small intestinal biopsies where histologic examination shows villous atrophy, crypt hyperplasia and intraepithelial lymphocytosis, and the presence of circulating CeD-specific antibodies to tissue transglutaminase (tTG), deamidated gliadin peptides (DGP), and endomysium (EMA). In children who have symptoms suggestive of CeD, a strongly positive tTG antibody (tTGA) titre and a CeD-associated HLA genotype, the diagnosis of CeD may be possible without the need for small intestinal biopsy (2). Since the 1950s when gluten was identified as the causative trigger of CeD, a strict and lifelong gluten-free diet (GFD) has been the mainstay of treatment.

While CeD is common around the globe and is rising in prevalence in many populations, it is frequently undetected in clinical practice (3). Symptomatic and untreated disease is associated with elevated morbidity and mortality and impaired quality of life (4–7). The clinical presentation is broad and includes gastrointestinal upset, chronic fatigue, nutrient deficiencies, poor growth, and failure to thrive. Extra-intestinal manifestations are common, and while they were once considered more frequent in adults than children with CeD, recent data suggests the frequency is similar in CeD children, although the types and rates of recovery differ (8). In children, extra-intestinal manifestations include short stature, anemia, delayed puberty, dental enamel hypoplasia, reduced bone density, oral ulcers, liver and biliary disease, and dermatitis herpetiformis. Poor growth and anemia tend to be the most common and there is a correlation with more severe histological damage at diagnosis compared to children with a gastrointestinal presentation or screen-detected cases (9). Insidious effects of undiagnosed CeD in children include behavioral disturbances and reduced educational performance (10).

CeD is also associated with an increased risk of autoimmune diseases including Hashimoto's thyroiditis, Graves' disease and type 1 diabetes (T1D) (11–14). A large Danish population study revealed the prevalence of autoimmune diseases was 16.4% among CeD patients compared with 5.3% in the general population in 2016 (12). Approximately 5% of patients with CeD have T1D and ~6% of T1D patients have CeD (15). In Northern Sardinia, a population with a high prevalence of CeD (16), patients with autoimmune thyroiditis had a 4-fold greater prevalence of CeD than the general population and while iron deficiency was present in almost half, none had gastrointestinal symptoms (17). The co-occurrence of autoimmune diseases supports the concept of shared genetic and immune pathways contributing to immune dysregulation and loss of self-tolerance, however it remains unclear whether CeD directly leads to other autoimmune disease and whether early diagnosis and treatment with a GFD alters this risk (18). Next generation sequencing of the HLA region shows that extended class II haplotypes differ between populations (19); this may partially explain regional differences in the degree of association between CeD and autoimmune disease. The strong association of CeD with autoimmunity, especially T1D and autoimmune thyroid disease, supports screening for these conditions in CeD patients.

The consumption of gluten containing foods is necessary for CeD to develop. Gluten is the viscoelastic protein that remains after washing dough and is composed of alcohol-soluble gliadins and alcohol-insoluble glutenins. The rheological properties of gluten allow it to impart a light and extensible texture to food making it highly favored in the food industry. Modern wheat gluten arises from a hexaploid genome, making it heterogeneous and more genetically complex than the human genome. Similar proteins rich in glutamine and proline (thus, the collective term prolamins) are found in barley and rye and termed hordeins and secalins, respectively, and are also toxic in CeD. Avenin, the prolamin component in oats, is phylogenetically distinct from wheat, barley and rye prolamin. Oats are considered safe for consumption by most people with CeD, although adverse immune and clinical effects have been reported (20–22) and some cultivars may be more immunogenic (23). Several expert reviews have concluded that further research into the toxicity of oats in CeD is needed (24, 25).

Why CeD develops in some people remains unanswered. Epidemiologic and prospective observational studies implicate a range of environmental factors that impact disease development. A role for the microbiome in the development of tolerance and disease pathogenesis is also emerging. Genetic and immunological studies have revealed the importance of key HLA and non-HLA susceptibility genes in disease development and a long-lived pathogenic population of gluten-specific T cells targeting certain gluten peptides (T cell epitopes). The emerging story of CeD development is one where environmental factors increase the risk of CeD in genetically predisposed individuals by shaping the immunologic context in which gluten is presented and shifting the balance from gluten tolerance to reactivity, and that this may be in part mediated through microbiome-host interactions. Contemporary clinical issues of importance include expediting the detection and diagnosis of CeD, improving and quality of life and health outcomes for those diagnosed, and developing treatments that are more effective and less burdensome than the current approach of a lifelong and strict GFD. This article will review current knowledge about CeD development and pathogenesis and how this knowledge is being applied to explore novel diagnostic, treatment and preventative approaches.

## The role of environment in disease development

CeD is a global disease that has been reported in Western and Eastern Europe, North America, South America, Asia, Oceania, and Africa (3, 26). It appears to be relatively uncommon in southeast Asia and sub-Saharan Africa. Recent reports from China suggest the illness could be substantially unrecognized there however more biopsy-based studies are required (27). In a systematic review and meta-analysis, the global seroprevalence and biopsy-confirmed prevalence of CeD was estimated to be 1.4% and 0.7%, respectively (3). The sero-prevalence of CeD in the US from National Health and Nutrition Examination Surveys (NHANES) was 0.7% and, consistent with a range of population studies from around the globe, showed that most cases remain undiagnosed in the community (28). Since CeD is frequently undiagnosed an active case-finding approach is considered best practice.

The prevalence of CeD varies with sex, age, and geographic location with the frequency of predisposing HLA haplotypes in the general population and per-capita wheat consumption the two major determinants of prevalence. There is a modest gender bias in favor of females (29). Familial clustering in CeD is common with 10% of first-degree relatives of a CeD sufferer affected. The high concordance rate for monozygotic twins (~80%) compared to HLA-identical siblings (~30%) and dizygotic twins (~10%) underscores the importance of both genetic factors (HLA and non-HLA genes) and the environment in CeD risk (30).

The high prevalence of CeD observed among populations living in areas with a high consumption of wheat products are highly suggestive for involvement of dietary gluten in CeD development (26). Although intake of gluten is necessary for CeD to develop, it does not solely explain why not all genetically-predisposed individuals consuming gluten develop CeD and why the disease can develop later in life despite many decades of gluten intake. Significant differences in the prevalence of CeD between people of similar genetic background and wheat intake living in nearby regions (for example, Finland and Russian Karelia) is strong evidence that the risk of CeD is influenced by other factors apart from genetic susceptibility and wheat consumption (31). Indeed, population studies have implicated a range of environmental factors associated with CeD risk (summarized in Table 1). Heterogeneity of study design has yielded conflicting results in the search for triggers in CeD. We have limited our review to focus on the association between CeD and diet, infections, antibiotic use, and delivery mode.

**Table 1:** Environmental factors potentially associated with CeD development.

**Risk factor**	**Effect on CeD risk**	**Studies (refs)**
**GLUTEN INGESTION**		
Age at gluten introduction (timing)	No association	Systemic review with meta-analysis (32); review (33); RCT (34).
Amount of gluten introduction	Conflicting data	A case-control study showed the amount of gluten consumed until 2 years of age increased CeD risk (35); An RCT in HLA-at risk infants with low dose of gluten (100 mg) introduced at 4–6 months showed overall no effect on risk (36).
**INFECTIONS**		
Infections (overall)	Increased	Increased risk of CeD especially with many infections (10 or more) up to 18 months of age (37).
Infections (gastrointestinal)	Increased	Gastrointestinal infection increased CeD risk autoimmunity by 33%. Risk was reduced in children vaccinated against rotavirus (38).
Rotavirus	Increased	In Sweden rotavirus vaccination has not reduced CeD prevalence (39).
Reovirus	Past infection associated with CeD and possible mechanism established	Higher prevalence of reovirus antibodies in CeD patients vs. controls; Reovirus infection may impair development of oral tolerance (40).
*Helicobacter pylori*	Conflicting data	Inverse relationship with CeD (41–43); positive or no association with CeD (44, 45).
**PERI-NATAL FACTORS**		
Season of birth	Increased risk if born in summer	Multiple populations assessed in different studies (38, 46–48).
Elective cesarean section	No association	Multiple populations assessed in different studies e.g., Norwegian Mother and Child (MoBa) Cohort Study (49), TEDDY cohort (50) and others (51).
Geographic location	Possibly increased with northern latitude (single study)	National Health and Nutrition Examination Survey (NHANES) database; CeD more common in northern compared to southern latitudes (52). However, exceptions to the north-south gradient exist e.g., high prevelance of CeD in Northern Africa (26), (53) and Sardinia (16).
Socio-economic status	Increased risk with higher SES	Unclear if due to biological effect e.g., hygiene hypothesis (31) or if due to differences in health seeking behavior (54).
Maternal gluten consumption	No association	TEDDY cohort; mother's intake of gluten in late pregnancy was not associated with risk of celiac disease in offspring (55).
**MEDICATIONS**		
Proton Pump Inhibitors (PPI)	Increased	Prior use of PPI strongly associated with CeD: OR 4.79; 95% CI 4.17-5.51) (56).
Antibiotics	No increased risk	Use of the most prescribed antibiotics during the first 4 years of life was not associated with the development of autoimmunity for T1D or CeD (57).
Maternal iron supplementation	Conflicting data	Increased risk in MoBa cohort (58) but not replicated in TEDDY cohort (59).
Vitamin D	No association	Maternal or neonatal vitamin D status not related to the risk of childhood CeD (60).

### Infant feeding

The steep rise in CeD incidence in young children after changes in the Swedish national infant feeding recommendation in the mid-1980s that suggested postponing gluten introduction from 4 to 6 months of age hinted that timing of gluten intake influenced CeD risk (61). However, the epidemic of CeD that occurred in Sweden occurred simultaneously with companies raising the gluten content in commercial baby formulas and was confounded by an observed protective effect of long breastfeeding duration (62). This made it difficult to disentangle whether timing or amount of gluten intake in relation to weaning impacted on the risk of CeD. The hypothesis that timing of first gluten exposure was associated with CeD was further supported in a study that found infants exposed to gluten either early (< 4 months) or late (>7 months) were at an increased risk (63). Since this first prospective study was published several follow-up papers from larger longitudinal prospective birth cohorts summarized in two recent systemic reviews with meta-analysis (32, 33) have not been able to confirm the previous findings that either age of gluten introduction or breast-feeding influence CeD risk.

Although there are wide differences in gluten intake between countries (64), it is not entirely clear whether the quantity of gluten intake during early childhood affects the risk of CeD. A Swedish retrospective case-control study indicated that children that later developed CeD consumed larger amounts of gluten before the age of 2 years than healthy children (62). This finding was in line with another cross-sectional study from the same group that observed a lower prevalence of CeD in a birth cohort reporting a lower gluten consumption in children born *after* (65) as compared to children born *during* the years of the Swedish epidemic (66). In a nested case-control study, a high intake of gluten amount increased the risk for CeD in Swedish children (35). However, whether gluten intake contributes to CeD development is still controversial as another multicenter study consisting of five other European countries found no association with CeD and gluten amount except for children carrying the lower-risk HLA-DQ2.2/DQ7 haplotype (67). Larger prospective studies with a longer follow-up are underway and will shed light on whether gluten intake is an independent risk factor in CeD.

### Infections

Several studies have shown that children that later develop CeD are more frequently affected by infections during early life (37, 68, 69). One limitation is that these studies are based on questionnaires filled in by parents and the type and site of infection is not specified. In a multicenter, prospective birth cohort study parents that reported a gastrointestinal infection 3 months prior to seroconversion of tTGA were at an increased risk of CeD autoimmunity later in life (38). There is also an effect of seasonality on the risk of developing CeD, hypothesized to be caused by viral infections occurring during a vulnerable period of immune development. This is supported by the association with frequent rotavirus infections and increased risk of CeD autoimmunity from longitudinal prospective studies (39) and a protective effect of rotavirus vaccination (38).

How infections trigger CeD development remains unexplained. Gastrointestinal infections may increase gastrointestinal permeability to increase the passage of gluten across the mucosa, or elevate tTG expression that can increase the generation of immunogenic gluten peptides. Molecular mimicry could possibly occur if the foreign antigen (such as a virus or bacteria) shares sequence or structural similarities with gluten itself and then initiates an anti-gluten response. Several studies have shown antibodies to adenovirus (70–72) and rotavirus peptides (73) circulating in CeD sera but further studies are required to determine the significance of these associations with disease pathogenesis. In recent work in mice, viral infection led to a break in oral tolerance to dietary proteins (40). Some reoviruses can promote a proinflammatory phenotype in mouse dendritic cells (DCs) which lose their capacity to promote tolerance toward food antigens and cause a pathogenic T cell response instead. Reovirus infection causes increased signaling by type 1 interferons and increased expression of the transcription factor interferon regulatory factor 1 (IRF1) which can block the conversion of T cells into regulatory T cells (Tregs) and promote a proinflammatory TH1 response to dietary antigens, respectively. Supporting relevance in humans, patients with CeD tended to have higher anti-reovirus antibody titers. Importantly, reovirus infections are often silent or asymptomatic in humans and a large proportion of the population is exposed to self-limiting gastrointestinal infections during childhood. The findings provide a mechanistic explanation that links an apparently innocuous virus with the loss of tolerance to a common food antigen. More research is required to unravel the significance of viral, bacterial or other microbial host interactions or infections in the development of CeD.

### Antibiotics and delivery mode

Early case-control studies reported a link between prior antibiotic use and subsequent CeD development in both adults (74) and children (69). Similarly, children with CeD were more likely to have been born by cesarean section (c-section) (75). A large case-control study found that while emergency c-section was not associated with later CeD development, elective cesarean delivery was (76). However, conflicting data has been reported. For instance, no link between increased CeD risk and antibiotic use during the first 6 months of life (68) or antibiotic use during pregnancy (77) was found. The Environmental Determinants of Diabetes in the Young (TEDDY) study is a multicenter observational cohort study that aims to identify environmental factors associated with T1D and CeD in children at HLA risk followed from birth (78). It found no association between antibiotic use and CeD autoimmunity during the first 4 years of life (57) or between delivery by c-section and increased CeD risk (50). Similarly, large observational studies found no link between c-section and CeD development (49, 69, 79). Finally, a large register-based study, that included children from two independent cohorts, found that birth delivery mode was not associated with increased risk of diagnosed CeD (51). Although the data is conflicting, the potential links between early events that can alter the microbiota composition, such as antibiotic use or birth delivery mode, and later CeD implicate a role of the microbiome in disease development.

## The microbiome in celiac disease

Microbial colonization occurs at birth and shapes the development of the mucosal and systemic immune system and the intestinal barrier. These host-microbe interactions continue throughout life, and a disruption of these interactions, through altered bacterial composition or functions, have been hypothesized to increase the risk of a range of autoimmune or inflammatory diseases such as CeD. Altered microbiota composition in patients with CeD may represent an environmental modifier of CeD development.

An early study described the presence of rod-shaped bacteria in duodenal biopsies of Swedish children with CeD born during the epidemic, which weren't observed in biopsies of control children (80), or in children born following the epidemic (81). The bacteria were subsequently identified as *Clostridium spp, Prevotella spp*, and *Actinomyces spp*, and their presence was suggested to be a risk factor for CeD that contributed to the increase in disease incidence in Sweden from 1985-1995 (81). Subsequent clinical studies have described differences in both fecal and duodenal microbial composition in children and adults with active compared with treated CeD, or healthy controls (82). While no specific microbial signature has been described for CeD, many groups have described increases in the proportions of *Bacteroides* and members of Proteobacteria, and decreases in *Lactobacillus* and *Bifidobacterium* (83, 84). In addition, CeD patients suffering from persistent symptoms were shown to have increased abundance of Proteobacteria compared to those who were asymptomatic (85). While these studies suggest an association between altered microbial composition and development of CeD, studies exploring mechanisms and causality are lacking. Moreover, whether alterations in the microbial composition are a cause or consequence of small intestinal inflammation has not been fully elucidated.

Recent studies have suggested that the microbiota from CeD patients may harbor more pathogenic or pro-inflammatory bacteria. Reports of CeD diagnosis following *Campylobacter jejuni* infection (86) suggest that bacterial infections could precede CeD development. *Escherichia coli* clones isolated from CeD patients expressed a higher number of virulent genes compared to those isolated from healthy controls (87). Similarly, the presence of virulent genes were higher in *Staphylococcus spp* and in *Bacteroides fragilis* strains isolated from CeD patients compared to healthy controls (88, 89). Importantly, strains isolated from CeD patients were more pro-inflammatory *in vitro* and stimulated altered DC morphology, characteristic of DC maturation, increased pro-inflammatory cytokine production, and altered epithelial barrier integrity. Similarly, *Neisseria flavescens*, a member of Proteobacteria, was identified in the duodenum of active CeD patients but not from control subjects and induced an inflammatory phenotype in human and murine DCs (90).

In contrast to the above studies, bacterial infections may also protect against CeD development. Some studies indicate an inverse relationship between the presence of *Helicobacter pylori* and CeD in both adults and children (41–43) whereas other studies have shown a positive or no association (44, 45). Mechanisms underlying this association have not been elucidated and inconsistencies across studies may relate to differences in techniques used to determine *H. pylori* status or *H. pylori* virulence. Less virulent strains may exacerbate the mucosal response in CeD whereas more virulent strains may provide protection against CeD (45, 91).

Functional differences in the microbiota could also affect metabolic processes important in CeD pathogenesis. The gastrointestinal tract harbors diverse bacteria that participate in gluten metabolism *in vitro* and this may differ between healthy individuals and those with CeD (92–94). As most studies have measured microbial composition in active or treated CeD compared to healthy controls it is difficult to determine whether functional differences are present prior to disease onset.

To gain insight into the potential role of microbial factors in CeD development, previous studies have profiled the fecal microbial composition of genetically at-risk children. High-risk children were shown to harbor a different microbiota compared to children who were at low genetic risk for CeD (95, 96), suggesting that the high-risk genotype may influence early gut microbiota composition. Infants at the highest risk for CeD had a higher prevalence of enterotoxigenic *E. coli* compared to those at low or intermediate risk for CeD (97). In addition, in a cohort of 164 infants, those at risk for CeD had lower numbers of *Bifidobacterium spp* and *B. longum* and increased numbers of *B. fragilis* and *Staphylococcus spp*. The differences in *Bacteroides* and bifidobacteria were attenuated by breastfeeding (98). At-risk children that later developed CeD were recently shown to have an altered microbial trajectory that coincided with immune changes. These changes were suggestive of a “premature maturation” of the gut microbiota in children who went on to develop CeD (99). On the other hand, the fecal microbiota of at-risk infants who went on to develop CeD was similar at 9–12 months to those infants that remained healthy by the age of four (100). Whether the duodenal microbial composition or function is altered in at-risk individuals that go on to develop CeD needs to be investigated further in larger clinical trials.

Diet and environment also determine gut microbiota composition (101, 102), highlighting the complexity of delineating the influence of genotype and environment on shaping the microbiota. Larger clinical trials where both the composition and function of the microbiota is studied in at-risk individuals and followed over time are needed to help understand gene-microbe interactions in CeD development.

## The role of genetics in disease development

While environmental factors are important for CeD development a notable feature of CeD is its high heritability and strong HLA association (103). This strong genetic association reflects the central role of CD4+ T cells as the HLA molecules associated with CeD bind specific gluten peptides that activate T cells (104). Ninety percent of Caucasian CeD patients possess the HLA-DQ2.5 haplotype (encoded by the DQA1^*^05:01 and DQB1^*^02:01 alleles) either in *cis* or *trans* positions, and the remaining carry either HLA-DQ8 (encoded by the DQA1^*^03:01 and DQB1^*^03:02 alleles), HLA-DQ2.2 alone (encoded by the DQB1^*^02:02 allele) or HLA-DQ7 alone (encoded by the DQA1^*^05:01 allele). Less than 1% of CeD patients lack these HLA haplotypes (105) and their absence can be exploited in the clinical setting to assist in excluding a diagnosis of CeD.

A “gene-dose effect” related to the number of copies of the DQB1^*^02 allele has been reported to affect CeD risk, clinical phenotype and patient responses to a T cell targeted therapy. The presumed basis for this effect is that gluten presented by APCs in HLA-DQ2.5 homozygous (i.e., two copies of DQB1^*^02) individuals can induce at least a 4-fold higher T-cell response compared with gluten presented by APCs in HLA-DQ2.5 heterozygous (i.e., one copy of DQB1^*^02) individuals (106). The CeD risk in HLA-DQ2.5 homozygous patients is ~2.5 and 5 times that conferred by HLA-DQ2.5 heterozygosity and lower risk HLA groups, respectively (107). A prospective Italian study (Celiac Disease and Age at Gluten Introduction study; CELIPREV) followed newborns with a family history of CeD and showed the risk of CeD autoimmunity (positive CeD-serology panel) at 10 years of age was far higher among children who were HLA-DQ2.5 homozygous (or who had two copies of DQB1^*^02 than among those who were HLA-DQ2.5 heterozygous or HLA-DQ8 (38 vs. 19%, *P* = 0.001), as was the risk of overt CeD (26 vs. 16%, *P* = 0.05) (34). In this cohort, 80% of those in whom CeD developed did so during the first 3 years of life. In the TEDDY study following 6403 US and European genetically at risk children at for CeD, the risks of CeD autoimmunity and confirmed CeD by age 5 were 11 and 3%, respectively in the heterozygous children and 26 and 11%, respectively, in those who were homozygous (108).

In addition, HLA-DQ2.5 homozygosity has been associated with a more severe CeD phenotype with earlier disease onset, greater villous atrophy, diarrhea, and lower hemoglobin at presentation, and a slower rate of villous healing on a GFD (109), plus a higher rate of refractory (non-responsive) CeD (110). In a recent clinical trial of an immunotherapy targeting gluten-specific CD4+ T cells, CeD subjects who were HLA-DQ2.5 homozygous were more likely to experience gastrointestinal symptoms following systemic administration compared to those who were heterozygous (111).

The contribution of non-HLA genes to CeD risk susceptibility is much less strong (OR < 1.5) than the HLA-associated haplotypes (OR >5) but collectively are significant. More than 70 candidate genes in over 40 non-HLA loci have been implicated in CeD heritability (112–117). These loci encode proteins involved in a range of immune pathways affecting T and B cell activation, chemokine receptor activity and cell migration, cytokine binding, thymic differentiation of CD4+ and CD8+ T cells, stress pathways and innate immunity. Only one gene has been shown to be gut specific (*RGS1*), underscoring the systemic nature of immune dysregulation in CeD (114). To date there is no evidence to implicate specific alleles encoding gastrointestinal proteases or tTG. Mirroring the frequent disease co-occurrence, there is substantial overlap between genetic risk factors for CeD and those of autoimmune diseases such as rheumatoid arthritis, multiple sclerosis and T1D (112, 115, 118, 119). Furthermore, despite a much weaker clinical association with CeD, there is overlap of genetic risk loci for inflammatory bowel disease such as Crohn's disease (18, 120). Intriguingly, 90% of the identified risk loci map to non-coding regions such as promoter regions, enhancers or non-coding RNA genes, suggesting that regulation of gene expression rather than changes at the protein-coding level are more important for CeD susceptibility and development (121).

## A key role for T cells in pathogenesis

**Figure 1:**
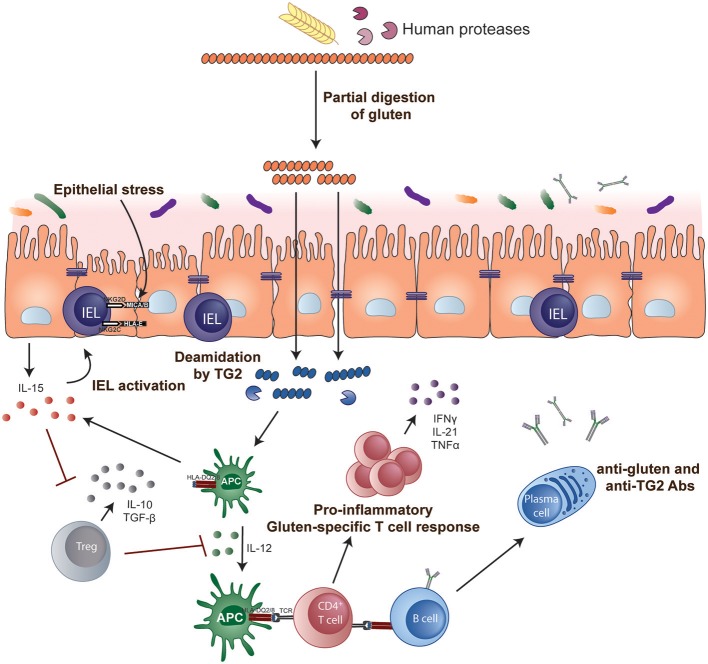
Key steps in CeD pathogenesis. Gluten peptides containing T-cell epitopes resist gastrointestinal degradation. tTG catalyses the deamidation of gluten peptides, which can then bind more efficiently to the disease-relevant HLA-DQ molecules on APCs. Activated gluten-specific CD4+ T cells secrete a variety of pro-inflammatory cytokines such as IFN-γ and IL-21 that contribute to the intestinal lesion and promote activation of IELs and stimulate B-cell responses. Activated IELs transform into cytolytic NK-like cells that mediate destruction of enterocytes expressing stress signals. IL-15 renders effector T cells resistant to the suppressive effects of Tregs and, in the lamina propria, endows mucosal DCs with inflammatory properties promoting pro-inflammatory responses and preventing Treg differentiation.

The role of CD4+ helper T cells in CeD was confirmed with the isolation of pro-inflammatory gluten-specific CD4+ T cells from intestinal tissue of CeD patients (122) (Figure 1). These pathogenic T cells have a Th1 phenotype characterized by production of IFN-γ and TNF-α (123) and almost all are HLA-DQ2- and/or DQ8-restricted (122, 124, 125). Gluten peptides that have been post-translationally modified by the enzyme tTG in a process called deamidation can effectively activate these T cells (126, 127). Deamidation converts specific glutamine residues to glutamate and this modification enhances the gluten peptide's binding affinity to disease-associated HLA dimers (128–130). Deamidation is crucial in converting poorly immunogenic wild-type gluten peptides to highly immunogenic antigens for CD4+ T cells. The structural requirements that generate effective binding of gluten peptides to HLA-DQ2 or DQ8 and T cells via the T cell receptor (TCR) have been further elucidated in structural studies (131, 132) and assessment of the biased use of TCR genes (133, 134).

Studies of T cells isolated from the intestine of CeD patients, or from their blood after short-term oral gluten challenge, have been used to define the gluten peptides (specifically, the T cell epitopes) immunogenic in CeD. Most studies have focused on the 90% of CeD patients who are HLA-DQ2.5 and a range of immunodominant T cell epitopes have been defined (135). Less is known about the nature of the T cell response to gluten in CeD patients without HLA-DQ2.5. The most immunogenic gluten epitopes for CeD patients with HLA-DQ2.5 after wheat ingestion reside in α-gliadin and ω-gliadin (136). Much of the field has focused on the immunogenicity of T cell epitopes in α-gliadin, specifically those encompassed within a protease resistant 33mer (137). However, gluten challenge studies show that the most immunogenic peptides induced by gluten ingestion depends on whether wheat, rye or barley is ingested, and that a sequence from ω-gliadin (encompassing the T cell epitopes DQ2.5-glia-ω1 and DQ2.5-glia-ω2) is dominant irrespective of which grain is consumed (136). Despite the multitude of immunogenic peptides, just three peptides from wheat and barley appear to recapitulate most of the response to gluten in CeD patients with HLA-DQ2.5. Interestingly, after oat ingestion, about 8% of CeD patients have detectable T cells specific for avenin peptides that share close sequence homology with barley hordein, suggesting that cross-reactive T cells may mediate immune responses following oats ingestion in some CeD patients (21).

While early work suggested T cells from children with CeD displayed a different pattern of reactivity to gluten compared to adults (138), more recent studies show gluten-specific T cells in blood induced by oral wheat challenge (139), or expanded from the small intestine during active disease (140), share the same specificity for deamidated, immunodominant T cell epitopes across all ages. The same gluten-specific T cell clonotypes persist in patients' blood and intestinal tissue up to several decades and share the same TCR gene use motifs in CeD patients from Norway, Finland and Australia (134, 139, 141, 142). Their stability over such long periods of time may be maintained by ongoing gluten exposure as inadvertent gluten intake is common in CeD even when a strict gluten-free diet is attempted (143, 144).

## Antibodies and B cells in celiac disease

Measurement of tTGA is a useful screening test for CeD as the titer reflects disease activity caused by gluten, however a direct role in disease pathogenesis is less clear. As tTGA are anti-angiogenic they may contribute to some extra-intestinal manifestations of CeD (145). As tTGA are detectable in intestinal tissue prior to the typical enteropathy of CeD and predict future disease onset, antibody production is likely to occur early in disease development (146). However, approximately 50% of children with positive CeD serology normalize their levels despite ongoing gluten exposure, suggesting that gluten immunity that leads to a pathogenic response is not necessarily fixed once it has commenced (147). Supporting this concept is the observation that almost 20% of adults with CeD diagnosed during childhood who elect to resume gluten intake have no evidence of active disease (148).

Production of tTGA appears reliant on T cells, as antibody formation to tTG and DGP is strictly dependent on the presence of CeD-associated HLA types as well as gluten. This supports the idea that tTG-specific B cells internalize tTG in complex with gluten peptides and present gluten-derived peptides to gluten-specific T cells, effectively amplifying the T cell response. These T cells then provide the required “help” to the B cell, resulting in production of tTGA and DGP antibodies (149, 150).

In active CeD, a large number of plasma cells can be found in the intestinal lesion and tTG-specific plasma cells made up a large proportion of them (5–25%) (151). There is much that still needs to be understood about how these antibody producing cells are selected and mature. tTG can form covalently linked multimers with itself that readily bind gluten peptides and can be taken up by tTG-specific BCR transduced cells and activate gluten-specific T cells with increased capacity compared to tTG monomers (152). Immunoglobulin expressed on B cells could act as substrates for tTG, in particular IgD, resulting in BCR/tTG cross-linking (153). As B cell epitopes are in close proximity to immunodominant T cell epitopes (154) and react to a higher degree to deamidated peptides (155) future work needs to examine the B cell and T cell interaction during the gluten-specific immune response.

## The development of intestinal villous atrophy

The events that culminate in the histological changes of CeD are incompletely understood. Gluten T cell epitopes cluster in regions of high proline making them resistant to the effect of gastrointestinal proteases (156). These peptides may pass across the epithelium via transcellular (157–159) and paracellular (receptor or antibody mediated) pathways (160). Increased epithelial permeability, a feature of active CeD, may be mediated by a direct effect of gliadin acting via the chemokine receptor CXCR3 in intestinal epithelial cells on tight junctions (160). DCs are presumed to play a major role in presentation of gluten peptides to CD4+ T cells (161), however little is known about their identity, where this presentation occurs, and the extent to which this role is undertaken by gluten-specific B cells. Activated CD4+ T cells produce large amounts of IFN-γ, that may induce cytotoxicity of intraepithelial lymphocytes (IELs), as well as IL-21, which plays a role in T-cell-dependent B cell responses (162). IL-17 producing CD4+ T cells have been reported in untreated CeD (163) but their role and that of IL-17A production in CeD pathogenesis is less clear (162, 164). IL-15 and IFN-α feature prominently in the inflamed tissue in CeD patients (165, 166). IELs are believed to play an important effector role in mediating destruction of enterocytes in CeD in a TCR-independent manner. When activated by stress signals on intestinal epithelial cells such as HLA-E and MIC-A (167), IELs express high levels of NK activating receptors such as NKG2D and CD94/NKG2C and adopt a cytolytic phenotype capable of destroying enterocytes (168). IL-15 plays a key role by upregulating the activating NKG2D receptor and acting as a co-stimulatory molecule. The effect is to ‘license' cytotoxic IELs with the ability to kill intestinal epithelial cells expressing the stress-induced MIC molecules. Adaptive immunity to gluten and epithelial stress where cytotoxic IELs have acquired an activated NK cell phenotype may both be required for villous atrophy to develop in CeD (169).

**Figure 2:**
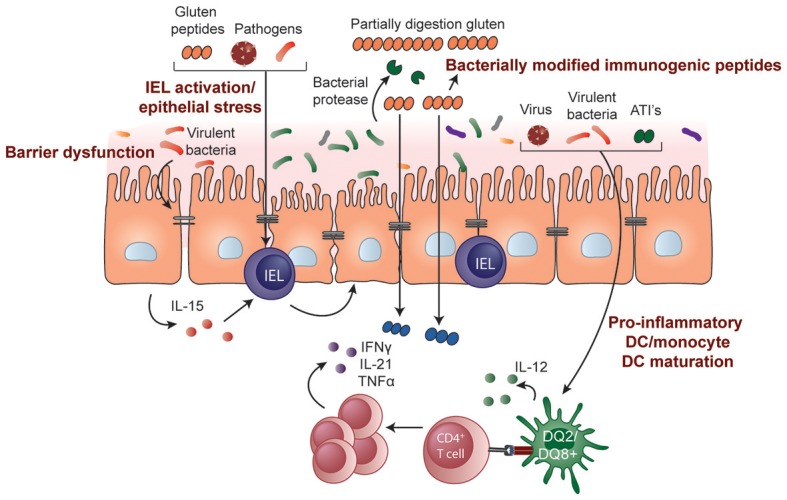
Potential role of microbes and environmental triggers in CeD pathogenesis. Microbes that include both commensals and opportunistic pathogens may contribute to the development of CeD by influencing gluten peptide digestion, intestinal barrier function, epithelial cell stress, or IEL activation/upregulation through IL-15 regulation. Pathogenic bacteria, viruses, and non-gluten components of wheat, such as amylase-trypsin inhibitors (ATIs), may also induce DC maturation and proinflammatory cytokine production, modulating the induction of CD4+ T-cell responses.

The causes of epithelial stress that trigger IEL activation and transformation in CeD are not known. Some hypothesize it is driven by gluten itself or other stimuli such as those resulting from a microbe-host interaction (Figure 2). The innate immune system is a pre-programmed form of host defense that responds rapidly to stimuli. Responses are triggered when pattern recognition receptors, for example, toll-like receptors on macrophages, bind molecules with conserved structures. An innate immune stimulatory effect of a gliadin sequence (A-gliadin p31-43) has been reported, but this work has not been replicated (170). No other gluten peptides activating innate immunity in humans have been defined. Gliadin may function as a stress signal for the activation of *MICA* expression only in the initial stages of disease and decline to baseline levels once the inflammatory lesion is established. In contrast to the critical role of HLA-restricted, gluten-specific CD4+ T cells in CeD pathogenesis, the relative contribution of innate immunity to disease has not been established in genetic or functional studies and further research in this area is required.

## How does loss of tolerance to gluten develop?

Although gluten consumption is common in the Western diet most individuals who possess HLA susceptibility for CeD develop immunologic tolerance to it as they do for other food proteins. Why a small percentage fail to develop or lose tolerance to gluten remains unresolved. Tregs are an important immune component contributing to intestinal homeostasis and when functioning normally inhibit pathogenic responses to dietary antigens and help maintain oral tolerance. This role suggests they may be important in CeD, however identifying and studying this cell population has been limited by the technical challenge of identifying a population of cells truly gluten-specific and functionally suppressive *in vivo*. These challenges have meant that studies of Treg function in CeD have been conflicting, with some indicating reduced suppressive function (171–173) and some showing it is retained (174, 175). Further studies that definitively isolate true gluten-specific Tregs and assess their function in CeD are needed.

Interestingly, the immunosuppressive cytokines IL-10 and TGF-β can be detected in high amounts in the CeD intestinal lesion but appear insufficient to counter active disease (176, 177). The high levels of pro-inflammatory cytokines such as IFN-γ and IL-15 may render this anti-inflammatory response inadequate (178, 179). IL-15 is a pleiotropic cytokine that may promote inflammation through several pathways, including driving the accumulation of cytotoxic IELs in the CeD lesion, interfering with the suppressive activity of Tregs (175), impairing TGF-β signaling (176), or allowing activation of disease-specific CD4+ T cells (180). IL-15 also drives the expansion of aberrant IEL clones that can lead to the development of enteropathy-associated T cell lymphoma. The multiple pro-inflammatory effects of IL-15 make it a rational target for therapeutic blockade, and clinical trials of anti-IL-15 biologic agents are underway in refractory CeD (181).

Additional insights into molecular pathways altered in loss of tolerance may be gained by study of gene expression profiles in CeD patients. Analysis of blood-derived non-gluten stimulated CD4+ T cells in CeD showed significant upregulation of the IFN-γ gene and reduced expression of a network of BACH2 regulated genes (182). BACH2 is a transcription factor that plays an important immunoregulatory role in inhibiting T effector cell development and promoting development of Tregs. Advances in techniques such as RNAseq now affords the opportunity to analyze multiple transcriptional and phenotypic features of antigen-specific effector T cells and Tregs to provide insights at the single cell level into these heterogeneous populations.

The findings on Treg numbers in the CeD lesion suggest that the defect in tolerance is not simply due to defects in numbers or recruitment of suppressive cells, but that the pro-inflammatory environment characteristic of CeD overcomes the tolerogenic milieu that normally maintains balance and inhibits abnormal immune responses. However, further studies on disease-relevant Tregs from the small intestine of CeD patients that assess antigen-specificity, function *in vivo* and the factors that impact their function are required.

## From pathogenesis to clinical care

### Insights from animal models

Modeling CeD has been a challenge as no single animal model that encompasses all elements of disease exists. As a result, mouse models have played a limited role in the development or pre-clinical testing of new therapies (183) and have more often been used to investigate specific mechanisms related to disease pathogenesis (184). The transfer of gluten-specific T cells into immunodeficient mice have been used to study the role of CD4+ T cells in mediating tissue damage (185, 186). Transgenic mouse models have also been used to investigate specific cytokines or genetic components in CeD pathogenesis. For example, mice over expressing IL-15 in the lamina propria (165) or in the epithelium (180) have shed light on the role of innate mediators in the development of the intestinal lesion in CeD. Mice that express human HLA-DQ2 or -DQ8 develop gluten-specific T cells and some innate immune activation following sensitization to gliadin with an adjuvant. However, they do not progress to full blown gluten-induced enteropathy (183, 187–189), emphasizing the importance of additional genetic, immune, or environmental factors in triggering tissue destruction in CeD. This lack of spontaneous loss of tolerance to gluten in transgenic mouse models can be taken advantage of and utilized to better understand environmental factors that participate in the loss of tolerance to gluten. For example, the mechanisms through which microbes contribute to CeD development can be studied by manipulating the microbiota composition or exposing transgenic mouse models to certain bacteria.

In mice expressing human HLA-DQ8 the composition of the gut microbiota was found to influence the degree of gluten-induced immunopathology (190). Mice harboring a limited microbiota devoid of Proteobacteria and opportunistic pathogens were protected from gluten-induced pathology and immune responses. However, this protective effect was lost when these mice were supplemented with an enteroadherent strain of *E. coli* that was isolated from a CeD patient. Similarly, treatment of specific pathogen free mice with vancomycin increased Proteobacteria levels, including *Escherichia*, and led to more severe gluten-induced pathology. While the mechanisms remain elusive, the results provide a proof-of-concept that microbes could alter how a host responds to gluten and could therefore be targeted as a prophylactic approach.

Gnotobiotic mice, or mice colonized with known microbes, provide a model where the impact of specific bacteria on gluten-mediated responses *in vivo* can be studied in a controlled environment. Studies of mice colonized with bacteria isolated from the duodenum of CeD patients or from healthy controls have shown that bacteria participate in gluten metabolism *in vivo* (94). Interestingly, the immunogenicity of the end products generated by bacterially-mediated gluten digestion differed depending on the type of bacteria. Following human protease digestion, elastase from *Pseudomonas aeruginosa* generated highly immunogenic gluten peptides that could strongly activate gluten-specific T cells from human CeD patients. These peptides were better able to translocate the epithelial barrier, potentially facilitating immune cell-peptide interactions. Conversely, gluten peptides produced following digestion by human proteases or by elastase from *P. aeruginosa* were detoxified or degraded by *Lactobacillus spp*, a core member of a healthy microbiome. The continued use of gnotobiotic models will be critical for understanding how microbes or pathogens may interact with the host and/or gluten to contribute to CeD pathogenesis (Figure 2). Importantly, these models can also be used to test microbiota-targeted therapies for CeD.

### Can celiac disease be prevented?

Population studies have provided important insights into environmental factors associated with CeD but are unable to establish true causality or mechanism. If factors that impact CeD risk can be identified and modified, then prevention of CeD may be possible. Randomized controlled trials (RCTs) do allow a controlled assessment of how a factor impacts CeD risk and several have now been undertaken or are underway in genetically at-risk infants or children (Table 2). The results have sometimes differed from assumptions made in population studies, underscoring the importance of running well designed and controlled intervention studies and undertaking research to examine mechanism.

**Table 2:** Prospective trials in infants/children looking at factors impacting celiac disease development.

**Study**	**Design**	**Finding**	**Reference or clinical trials identifier**
Prevent Coeliac Disease Study (PreventCD)	International double-blind placebo controlled RCT: 100 mg of gluten daily or placebo from 16 to 24 weeks of age	Neither breast-feeding nor introduction of small quantities of gluten at 16–24 weeks of age reduced the risk of celiac disease by 3 years of age in this group of high-risk children	(36)
Celiac Prevention Study (CELIPREV)	Multicenter RCT: Compare introduction of gluten at 6 vs. 12 months	Neither the delayed introduction of gluten nor breast-feeding modified the risk of celiac disease among at-risk infants	(26)
Celiac Disease Prevention With Probiotics Study (CiPP)	Double-blind RCT: Probiotic (*Lactobacillus*) vs. placebo in infants/children aged 2 to 12 years	Completed	NCT03176095
PreCiSe study	RCT: Probiotic vs. placebo vs. GFD from before age of 4 months for 3 years	In progress	NCT03562221

Two independent RCTs assessed whether low amounts of gluten can prevent genetically at-risk children from developing CeD. The idea of a “window of opportunity” during which gluten could be introduced in small amounts to induce tolerance was based on previous experience from the Swedish epidemic and supported by a prospective study showing infants exposed to gluten either early (< 4 months) or late (>7 months) were at an increased CeD risk (63). The optimal window was proposed between 4 and 6 months, preferably during ongoing breast-feeding (191). In the multicenter PreventCD study, almost 1,000 genetically at-risk children with at least one first-degree relative with CeD were randomized to a double-blind, placebo-controlled dietary-intervention to receive 100 mg of gluten daily or placebo from 16 to 24 weeks of age (36). Neither breast-feeding nor the introduction of small quantities of gluten at 16–24 weeks of age reduced the risk of CeD by 3 years of age in this group of high-risk children (36). Published in the same journal, the CELIPREV group randomised over 800 newborns who had a first-degree relative with CeD to have dietary gluten introduced at either 6 or 12 months (34). The results from the CELIPREV study concorded with the results from the PreventCD study, showing that neither the delayed introduction of gluten nor breast-feeding modified the risk of CeD among at-risk infants (34). Although CeD was not prevented by delaying the introduction of gluten, it was associated with a delayed onset of disease (34). Since these two RCTs were published, other clinical trials have been performed or are currently being conducted.

Another potential approach to CeD prevention is through modification of the host-microbe interactions in at-risk individuals. Trials to assess the impact of probiotic supplementation in preventing CeD in genetically at-risk children are underway (Table 2). If successful, they would support the idea that altered function of the microbiome is a major event underpinning the development of CeD, and that preventing dysregulated host-microbe interactions may be of prophylactic benefit. Furthermore, a better understanding of the mechanisms through which microbes contribute to CeD development can provide further rationale and a more targeted approach for microbiota-modulating preventative strategies.

### Improving the diagnosis of celiac disease

While villous atrophy remains the cornerstone of CeD diagnosis there is the growing realization that this “gold-standard” has limitations. For example, results are affected by the number of samples collected and how the biopsies are oriented and reported (192–195). Ultra-short CeD where villous atrophy is present only in the duodenal bulb and “mild enteropathy CeD” where villous atrophy is absent in the setting of positive CeD serology both present diagnostic challenges and highlight potential shortcomings of histology (196, 197). Improvement in the quality of serological testing for CeD and the requirement for specific HLA genotypes for CeD to develop has meant that a serogenetic approach to CeD diagnosis is appealing and may be sufficient for diagnosis in the right clinical situations (2, 198). As expeditious treatment of CeD may avoid or reduce the risk of many CeD-associated complications such as impaired bone density and stunted growth in children, improving early diagnosis remains a clinical and research priority.

In recent years the high rate of community adoption of the GFD, including in children (199), has compounded the challenge of CeD diagnosis as the accuracy of current serological and histological approaches depend on active gluten intake. In order to make a CeD diagnosis reintroduction of dietary gluten, generally for several weeks to months, is recommended prior to testing but patients are often reluctant to undertake this and for those that do many fail to tolerate it. As the serologic and histologic response to gluten challenge is highly heterogeneous the optimal duration of gluten challenge required for definitive diagnosis of CeD remains uncertain (200–202). Immune diagnostics that measure the gluten-specific immune response target a fundamental component of CeD and may overcome the limitations of current diagnostics. The use of tetramers (203) or cytokine release assays (204) to identify gluten-specific T cells induced in blood after short-term oral gluten challenge is highly sensitive and specific for CeD (205). Diagnostics that are accurate with limited or even no gluten exposure such as tetramer-based detection of gluten-specfic T cells (206) are particularly appealing to clinicians and patients as they may avoid the need for prolonged gluten challenge prior to testing with serology and histology. Large multi-center validation studies to confirm the accuracy of assessing disease-specific T cells as a CeD diagnostic are required, and if successful, may force a re-think of how CeD should be classified. Arguably, CeD may be better defined by the HLA-linked, T cell mediated systemic response to gluten rather than histologic changes in the proximal small intestine or circulating antibodies that indirectly reflect disease activity.

### Improving the treatment of celiac disease

While adherence to a strict and lifelong GFD still remains the single proven and available treatment for CeD, it is for many patients complicated, onerous, and expensive. In adults with CeD, daily consumption of as little as 50 mg of gluten, equivalent to that contained in 1/100th of a slice of standard wheat bread, over three months can damage the small intestine (207). A safe gluten “dose” threshold relevant to children with CeD has not been assessed in a controlled trial. Several longitudinal studies in adults with CeD indicate that failure to achieve mucosal healing is common even in those appearing to maintain good dietary adherence over many years (208–213). While healing is considered to be more complete and faster in children with CeD treated with a GFD one study showed 19% had persistent disease activity after 12 months on a GFD (214). Assuming enough time has elapsed on the GFD, persistent mucosal activity may be driven by ongoing, potentially intermittent, gluten exposure (143), such as that inadvertently consumed in contaminated meals when eating out (144). The challenge in maintaining adequately strict gluten exclusion and persistent disease activity is a major driver for research into new therapeutic approaches. While several therapies are under development it is notable that none of them have yet been evaluated in children.

Clinical trials of novel therapies for CeD have increased substantially in recent years but compared to other illnesses such as inflammatory bowel disease the field is still in its infancy. No therapeutic approach for CeD has yet completed Phase 3 clinical trials. An understanding of the optimal goals of treatment and the methods to assess efficacy are an evolving area and have been shaped by the requirements of regulatory bodies such as the FDA. Symptom improvement is now regarded as a key outcome measure and this has driven interest in validating patient reported outcome measures (215) and understanding the basis for gluten-induced symptoms in CeD. A standardized approach to reporting small intestinal histology based on quantitative assessment of morphology (villus height, crypt depth and their ratio) and inflammation (density of intraepithelial lymphocytes) is now commonly employed in CeD clinical trials (192). Confirming adequate dietary gluten exclusion during studies is a major challenge as symptom records, serology, histology, and dietary history are indirect measures of GFD adherence (216). New technology based on the detection of urinary or fecal gluten immunogenic peptides (GIPs) derived from the 33mer peptide in wheat α-gliadin provides objective evidence of dietary gluten exposure (217, 218). In addition to a role in the clinic, it may be a promising tool for evaluating and selecting patients for CeD clinical trials where controlling for inadvertent gluten exposure is important, such as therapies designed to prevent symptoms due to inadvertent gluten exposure (215).

Insight into the molecular mechanisms underpinning CeD pathogenesis provide several opportunities for novel therapeutics development and a range of pharmaceuticals are currently being assessed in pre-clinical and clinical trials (Table 3). These can be broadly classified into luminal approaches that aim to quantitatively reduce the load of gluten available to trigger the immune response and qualitative approaches that aim to induce gluten tolerance. A third category, not discussed in this review, encompass immunomodulators (e.g., budesonide, azathioprine), biologics (e.g., anti-IL-15, anti-CD52), and chemotherapy (e.g., cladribine) used to treat refractory CeD (219).

**Table 3:** Experimental therapies for celiac disease in pre-clinical or clinical development.

**Approach**	**Proposed mechanism**	**Phase of development**
**LUMINAL**		
Endopeptidases e.g., latiglutenase, An-PEP	Enzymatic degradation of gluten	Phase 2
Tight junction modulators e.g., larazotide acetate (AT-1001)	Reduce paracellular passage of gluten across mucosa	Phase 2
Transglutaminase inhibitors e.g., ZED 1227	Inhibit conversion of gluten to more immunogenic form	Phase 2
Gluten binding agents e.g., BL-7010	Sequester gluten in the intestinal lumen	Phase 1
HLA-DQ2 blockers	Prevent activation of gluten-specific T cells	Pre-clinical
Non-toxic gluten	Modified or selectively bred cereals devoid of toxicity	Pre-clinical
Inhibition of inflammatory proteases e.g., elafin	Anti-inflammatory effects and improved barrier function	Pre-clinical
**TOLEROGENIC**		
Peptide-based therapeutic vaccine (Nexvax2)	Epitope-specific targeting of gluten-specific CD4+ T cells	Phase 2
Hookworm (*Necator americanus*)	Immunoregulatory effect of hookworm combined with low-dose gluten exposure	Phase 2
Nanoparticle therapy (TIMP-GLIA)	Nanoparticle encapsulating gliadin delivered intravenously	Phase 1

Quantitative approaches include the use of (i) endopeptidase enzymes (glutenases) derived from plants, bacteria or fungi that have a gluten degrading effect, such as latiglutenase (ALV003) (220, 221) and AN-PEP (222) (ii) agents to reduce paracellular passage of gluten i.e., larazotide acetate, an intestinal tight junction regulator that may enhance barrier function (223–225), and (iii) compounds that bind gluten in the gut lumen to reduce absorption, such as the polymer BL-7010 (226). Supplements to the GFD which render small amounts of dietary gluten harmless could substantially improve the quality of life of patients by allowing them to dine out with less fear of adverse effects resulting from contamination by small amounts of gluten. Enzymatic approaches could also be applied during the baking process to reduce gluten immunogenicity (227). Genetic modification of wheat with a variety of targeted techniques such as RNA interference (228) and CRISPR (229) can reduce gluten T cell epitope content and immunogenicity however clinical feeding trials are awaited. The recent publication of the first fully annotated reference wheat genome is an important advance that may help guide targeted approaches (230). Use of protease inhibitors, such as elafin, which is decreased in the mucosa of patients with active CeD has been proposed as it has barrier enhancing and anti-inflammatory effects in gluten-sensitive mice (231).

A phase 2 RCT of latiglutenase taken orally showed it could attenuate small intestinal mucosal injury in CeD patients induced by 2 g of ingested gluten (221). In symptomatic CeD patients following a GFD, latiglutenase reduced symptoms in the subgroup who were seropositive (232), suggesting that gluten exposure was necessary in order to demonstrate a positive effect of the enzyme. In a Phase 2 trial larazotide acetate was shown to reduce symptoms in CeD patients on a GFD better than a GFD alone, but only at the lowest dose of 0.5 mg (225). More studies are required to establish the efficacy of these approaches and how they can be safely used by patients. A controlled gluten challenge will be an important component of the study design in order to demonstrate efficacy and establish the amount of ingested gluten patients can be protected from.

Qualitative approaches aim to establish durable immune tolerance to gluten. One way this might be achieved is by targeting the long-lived population of gluten-specific T cells and deleting or rendering them functionally unresponsive (anergic) and inducing suppressive Tregs (233). As the target T cell population is stable in established CeD (139, 140, 142), it is anticipated these approaches will apply similarly to children as they do in adults with CeD. Phase 1 studies of Nexvax2, a therapeutic vaccine composed of three gluten peptides encompassing immunodominant HLA-DQ2.5-restricted T cell epitopes, initially caused gastrointestinal symptoms similar to those triggered by gluten, however after later administration of Nexvax2 symptoms were no different from those after placebo (111, 234). The recall immune response to gluten was modified in people with CeD receiving Nexvax2. A phase 2 clinical trial of infection with the hookworm *Necator Americanus* combined with a micro-gluten challenge in 12 CeD patients showed immune modifying effects and clinical protection against gluten (235) and a larger controlled study is underway.

## Conclusion and future perspectives

Our view of CeD has evolved from a gastrointestinal illness to an immune disease characterized by the presence of specific HLA genes, CD4+ T cells responding to specific gluten peptides, circulating antibodies to tTG and systemic clinical manifestations. Aside from the fact it is driven by an exogenous, dietary antigen, the genetic and immunologic basis for CeD overlaps with that of traditional autoimmune diseases. While HLA susceptibility and wheat consumption and are major determinants of disease development it is apparent that non-HLA genes and a range of environmental factors are important for disease development. Prospective studies have established that the timing of gluten introduction and breastfeeding do not impact the development of CeD. More results from multicenter, prospective longitudinal studies are needed to understand the long-term effects of a high amount of gluten intake and to identify if other environmental exposures might trigger the disease.

Furthermore, although *in vitro* and *in vivo* studies suggests there are host-microbe or gluten-microbe interactions that promote gluten-specific immune responses, larger clinical trials where both the composition and function of the microbiota is studied in at-risk individuals and followed over time are needed to help understand gene-microbe interactions in CeD development. These kinds of studies may provide insight into the microbial events leading to CeD development that could be targeted as preventative or therapeutic strategies.

Finally, understanding how tolerance to gluten is lost in CeD is a fundamental question that needs more study. Insights into disease relevant pathways may come from analysis of the genome and gene expression in CeD, and epigenetic studies are needed to examine the impact of environment on gene expression and disease development. While novel therapies for CeD have not yet been tested in children, emerging studies on the role of environmental factors and the microbiome and how they might impact gluten immunity and tolerance may one day make disease prevention possible. For now, as primary prevention of CeD is a highly attractive, but as yet unrealized goal, the focus must be on driving expeditious diagnosis and treatment in symptomatic children and adults.

## Author contributions

JT-D wrote the first draft of the manuscript. DA and HG wrote sections of the manuscript. All authors contributed to manuscript revision, read, and approved the submitted version.

### Conflict of interest statement

JT-D has served as a consultant and scientific advisory board member for ImmusanT Inc., USA, and owns shares in Nexpep Pty Ltd and is a co-inventor of patents pertaining to the use of gluten peptides in CeD therapeutics, diagnostics and nontoxic gluten. Nexpep Pty. Ltd. and ImmusanT Inc. were formed to develop novel diagnostics and treatments for CeD. DA has served as principal investigator for probiotic studies in collaboration with Probi AB, Lund, Sweden, and is the co-inventor of patents pertaining to the use of *L. plantarum* (strain HEAL9) and *L. paracasei* (strain 8700:2) in CeD. The remaining authors declare that the research was conducted in the absence of any commercial or financial relationships that could be construed as a potential conflict of interest.
